# Supplementary material for: First Glimpse on Spring Starflower Domestication
Source: Genes (Basel). 2022 Jan 27;13(2):243. doi: 10.3390/genes13020243 (PMC8872604; doi:10.3390/genes13020243)
Supplement: Supplementary file 1 [file genes-13-00243-s001.zip › genes-1559959-supplementary.pdf]

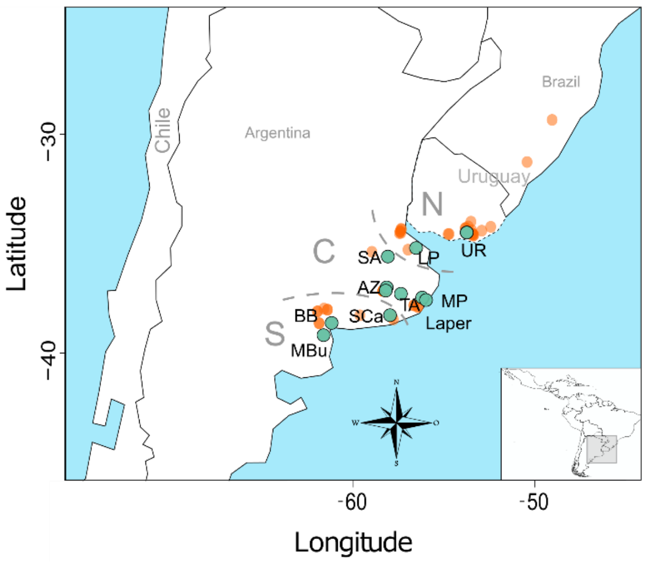

Figure S1. Map showing the geographic distribution of the wild populations of *Ipheion uniflorum* used in this study (green dots) and natural distribution of the species (orange dots). N: North; C: Centre; S: South.

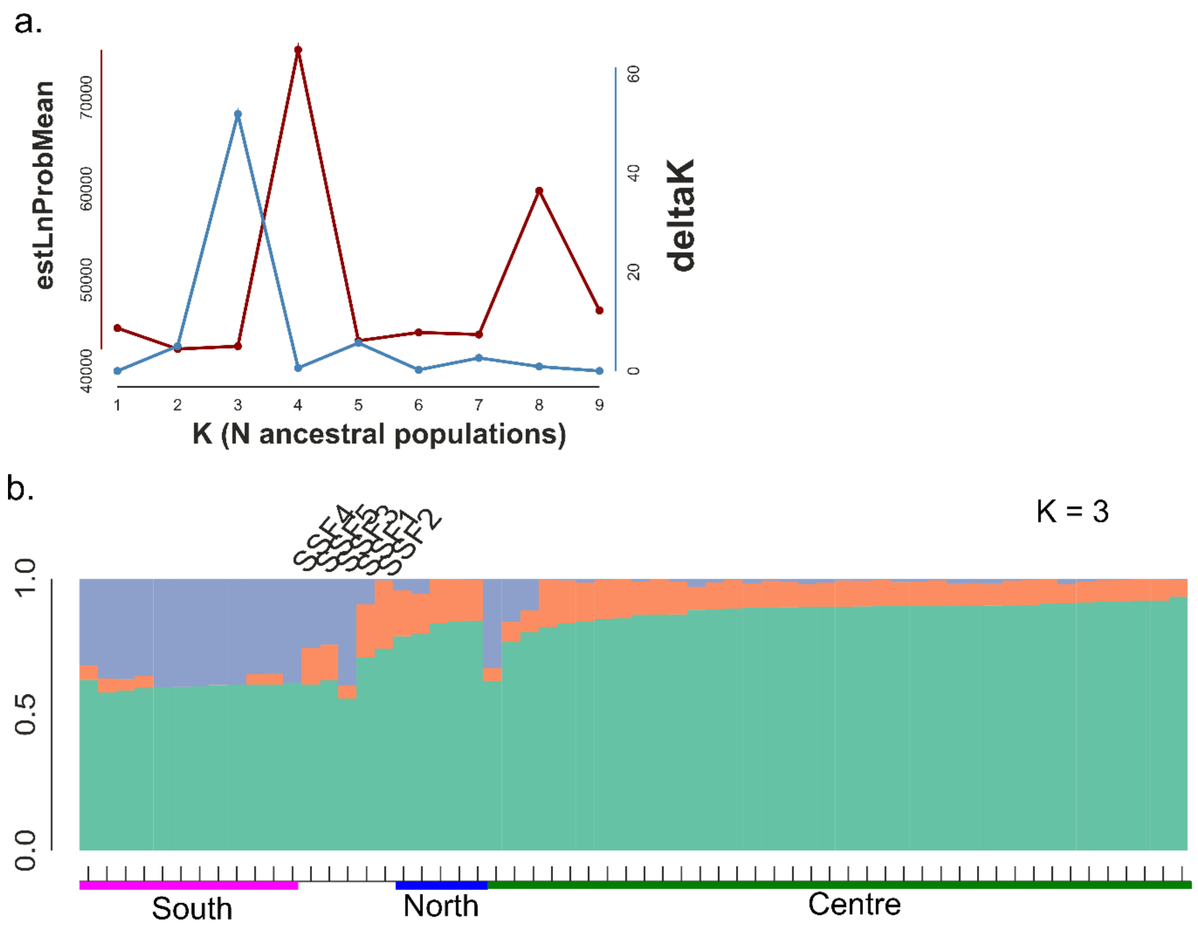

Figure S2. Population structure analysis. (**a**) Evanno's plot of K (number of genetic clusters) against corresponding mean estimated log-normal (Ln) probability of the data (dark red dots) and ΔK (light blue dots); (**b**) Population structure analysis of wild + cultivated *Ipheion uniflorum* specimens (n=61) based on K= 3 and 2302 unlinked SNPs. Each bar represents one individuals and colored segments represent the relative memberships to each genetic cluster.

Table S1. Subsampling of the morphological data.

Specimen	Region	Tepal apex	Flower colour	Floral tube length	Outer tepal width	Inner tepal width	Outer tepal length	Inner tepal length
502_uniMP_a	Centre	1	0	1	0.52	0.32	1.9	1.8
502_uniMP_b	Centre	0	0	1.6			2.1	
502_uniMP_c	Centre	0	0	0.9	0.48	0.36	2.2	2
510_uniLP_a	North	0	0	1.1	0.48	0.4	1.9	2
510_uniLP_b	North	0	0	1.1	0.504	0.44	2.1	2.1
510_uniLP_c	North	0	0	1.3	0.808		2.4	
513_uniAZ_a	Centre	0	1	1.2	0.408	0.336	1.8	1.6
513_uniAZ_b	Centre	0	1	1.1	0.5	0.4	2.6	2.6
513_uniAZ_c	Centre	0	0	1.1	0.576	0.496	2.5	2.3
540_uniAZ_d	Centre	0	1	1.1	0.648	0.576	2.4	2.2
540_uniAZ_a	Centre	0	0	1.2	0.6	0.4	2.4	2.3
540_uniAZ_b	Centre	1	0	2.1	0.44	0.6	2.4	2.5
540_uniAZ_c	Centre	0	0	1.4	0.44	0.36	2.3	2.2
540_uniAZ_d	Centre	0	0	2.5	0.6	0.4	2.4	2.2
541_uniTA_a	Centre	0	0	1.5	0.68	0.56	2.6	2.7
541_uniTA_b	Centre	0	0	1	0.48	0.511	2.3	2.1
541_uniTA_c	Centre	0	0	1	0.6	0.7	2.6	2.6
551_uniTA_a	Centre	0	0	1.2	0.64	0.48	2.6	2.5
551_uniTA_b	Centre	0	0	1.5	0.416	0.336	2.4	2.5
551_uniTA_c	Centre	0	0	1.2	0.68	0.44	1.6	1.5
551_uniTA_d	Centre	0	0	1.1				
551_uniTA_e	Centre	1	0	1.1	0.68	0.6	2.3	2.2
555_uniTA_a	Centre	0	0	1.3	0.816	0.816	2.6	2.5
555_uniTA_b	Centre	0	0	1.2	0.5	0.6	2.7	3
555_uniTA_c	Centre	0	1	1.3	0.5	0.6	2.6	2.5
555_uniTA_d	Centre	0	2	1.3	0.4	0.32	2.2	2.3
567_uniLaper_a	Centre	0	0	1.2	0.32	0.36	2.2	2.3
567_uniLaper_b	Centre	0	0	1.6	0.4	0.44	2.8	2.7
567_uniLaper_c	Centre	0	0	1.3	0.4	0.36	2	1.9
567_uniLaper_d	Centre	1	0	1.2	0.44	0.32	1.6	1.6
567_uniLaper_a	Centre	1	0	1.1	0.56	0.48	2.5	2.4
567_uniLaper_b	Centre	0	0	1.1	0.464	0.48	2.1	2.1
uniUR	North	0	0	1.2	0.576	0.44	2.2	2.1
uniSA_a	Centre	0	0	1	0.8	0.48	2.4	2.6
uniSA_b	Centre	0	1	2.2	0.88	0.52	2.3	2.6
uniSA_c	Centre	0	0	1	0.44	0.6	2.3	2.6

Codification: Tepal apex: 0: obtuse, 1: acute; 2: round. Flower color: 0: white; 1: blue/light blue; 2: pink or lilac; 3: violet.

Table S2. Summary of quantitative and qualitative morphological data portraying the variation found among natural and cultivated specimens of *Ipheion uniflorum*.

Condition	Tepal apex	Flower colour	Tepal width	Tepal length	Plant size
natural	0/1/2	0/1/2	0.3-1.1 cm	2-3 cm	6-20 cm
cultivated	0/1/2	0/1/2/3	0.5-1.1 cm	2-4 cm	7-30(40) cm

Data from natural populations: [9, 10]. Data from cultivated samples: [11, 12] and descriptive information provided by shops offering bulbs of SSF. Character codification follows Sassone et al. [19].
